# Syphilitic Epididymitis

**Published:** 1894-06

**Authors:** Geo. W. Davis

**Affiliations:** Professor of Genito-Urinary, Venereal and Skin diseases, University Medical College, Kansas City, Mo.


For Texas Medical Journal.

### SYPHILITIC EPIDIDYMITIS.

BY GEO. W. DAVIS, M. D.,
Professor of Genito-Urinary, Venereal and Skin diseases, University Medical College, Kansas City, Mo.

PETER S., age 26, single, laborer, born in Germany, has always been healthy. Negative history of injury, and denial of previous venereal trouble. No evidence of lung disease.

In the early part of last October, he noticed an ulcer on the dorsal surface of the prepuce, which was diagnosed and treated as a chancre. About one month after the first appearance of the sore, patient accidently discovered a small lump just above the left testis.

Previous to my attention being called to the case, he was seen by several physicians, and the enlargement pronounced a "malignant growth;" and then again it was supposed that it might be a hernia from omental protrusion.

February 16, about four months after the first appearance of the preputial ulcer, the patient came under my observation. On examination, I noticed the cicatrix of the ulcer, papulo-pustular eruptions, enlarged gland at the angle of the jaw, alopecia, headache, and the symptoms of secondary syphilis, generally. On examining the testes, found them normal, but discovered in the region of the globus major of the left epididymis, and in fact involving all of the epididymis and extending along the cord to the pubic bone, an enlargement, indurated and almost catilaginous to the feel.

As near as could be determined, this enlargement was about three and a half inches long by one inch in width, and obviously not attached to the pubic bone, but seemingly nearly filling the opening of the external abdominal ring. This swelling was indolent, and only a slight amount of pain was caused by manipulation. Rectal examination showed some tenderness.

Placed the patient cn anti-syphilitic treatment, pil. hydrarg., gr. i, three times daily, and eleven days from the commencement of this treatment was gratified to find the tumor much smaller and the veins more distinct.

Regarding the local treatment as unnecessary, I yet occasionally applied ung. hydrarg., to amuse the patient and quiet his imagination.

Recently the swelling was examined, and found to have disappeared almost entirely, the result of treatment thus confirming the diagnosis.

Syphilitic epididymitis was first described in 1863, by Dron, of France. The literature of the subject is limited, and not altogether satisfactory. The best authorities speak of the disease as of rare occurrence, and unanimously state that it does not soften or show signs of degeneration. This fact is controverted (so far as I am aware) in only one instance, and that by a case reported in the *New York Medical Record* for 1877, page 194.

The history there given is not, to my mind, proof conclusive that the case was one of syphilitic epididymitis, but probably gonorrhœal epididymitis occurring in a syphilitic subject, as the recorded facts show the patient with tight urethral stricture complicated by urinary retention and two attacks of gonorrhœal epididymitis, before he became syphilitic. This is the only record I can find of a reported autopsy.

The case I have reported is one of exceptional interest. In the examination of several hundred cases of scrotal tumors, I have never met one like it. The special features are its affecting only one side, while both sides usually are involved; its large size; but most remarkable of all, was its involving the cord. This exception is rare indeed, and only a single reference is all I can find of such an involvement.

